# Supplementary material for: Cation Charge as a Tool to Change Dimensionality in Organic–Inorganic Hybrids Based on Copper Thiocyanate Templated by 1,4-Diazabicyclo[2.2.2]octane
Source: Molecules. 2023 Apr 20;28(8):3608. doi: 10.3390/molecules28083608 (PMC10198343; doi:10.3390/molecules28083608)
Supplement: Supplementary file 1 [file molecules-28-03608-s001.zip › molecules-2349682-supplementary.pdf]

Electronic Supporting Information

# Cation charge as a tool for changing dimensionality of copper(I) thiocyanate based organic-inorganic hybride compounds

*Evgeny A. Goreshnik*, Svitlana R. Petrusenko*

Department of Inorganic Chemistry and Technology, Jožef Stefan Institute, Jamova 39 1000, Ljubljana, Slovenia

evgeny.goreshnik@ijs.si

**Table S1**. Selected geometrical parameters (distances/Å and angles/°) for **1**.

| | | | |
|---|---|---|---|
| Cu1—Cu2$^{i}$ | 2.8793 (17) | N5—C7 | 1.494 (17) |
| Cu1—S1 | 2.330 (4) | N5—C8 | 1.495 (16) |
| Cu1—S2 | 2.420 (4) | N5—C9 | 1.493 (17) |
| Cu1—S3$^{ii}$ | 2.432 (4) | C4—C8 | 1.54 (2) |
| Cu1—N3 | 1.946 (13) | C5—C7 | 1.54 (2) |
| Cu2—S2$^{ii}$ | 2.415 (4) | S1—C1 | 1.649 (12) |
| Cu2—S3$^{iii}$ | 2.477 (4) | S2—C2 | 1.703 (15) |
| Cu2—N1$^{iv}$ | 1.965 (11) | S3—C3 | 1.659 (14) |
| Cu2—N2 | 1.965 (13) | N1—C1 | 1.157 (16) |
| N2—C2 | 1.095 (19) | N4—C5 | 1.474 (19) |
| N3—C3 | 1.151 (19) | N4—C6 | 1.482 (18) |
| N4—C4 | 1.464 (19) | N5—H5 | 1.0000 |

| | | | |
|---|---|---|---|
| S1—Cu1—Cu2$^{i}$ | 133.05 (11) | S2$^{ii}$—Cu2—Cu1$^{ii}$ | 53.53 (11) |
| S1—Cu1—S2 | 103.62 (13) | S2$^{ii}$—Cu2—S3$^{iii}$ | 102.68 (13) |
| S1—Cu1—S3$^{ii}$ | 106.76 (14) | S3$^{iii}$—Cu2—Cu1$^{ii}$ | 53.36 (10) |
| S2—Cu1—Cu2$^{i}$ | 53.37 (10) | N1$^{iv}$—Cu2—Cu1$^{ii}$ | 137.8 (3) |
| S2—Cu1—S3$^{ii}$ | 103.88 (13) | N1$^{iv}$—Cu2—S2$^{ii}$ | 108.5 (4) |
| S3$^{ii}$—Cu1—Cu2$^{i}$ | 54.82 (10) | N1$^{iv}$—Cu2—S3$^{iii}$ | 108.2 (4) |
| N3—Cu1—Cu2$^{i}$ | 103.1 (4) | N1$^{iv}$—Cu2—N2 | 122.5 (5) |
| N3—Cu1—S1 | 123.6 (4) | N2—Cu2—Cu1$^{ii}$ | 99.7 (4) |
| N3—Cu1—S2 | 107.0 (4) | N2—Cu2—S2$^{ii}$ | 107.3 (4) |
| N3—Cu1—S3$^{ii}$ | 110.1 (4) | N2—Cu2—S3$^{iii}$ | 106.0 (4) |

Symmetry codes: (i) x, −y+1, z−1/2; (ii) x, −y+1, z+1/2; (iii) x, y, z+1; (iv) x−1/2, −y+3/2, z+1/2; (v) x, y, z−1; (vi) x+1/2, −y+3/2, z−1/2.

**Table S2**. Selected geometrical parameters (distances/Å and angles/°) for **2**.

| | | | |
|---|---|---|---|
| Cu1—S1$^{i}$ | 2.2304 (7) | N2—C2 | 1.4976 (17) |
| Cu1—S1 | 2.2304 (7) | N2—C2$^{iii}$ | 1.4976 (17) |
| Cu1—S1$^{ii}$ | 2.2304 (7) | N2—C2$^{iv}$ | 1.4976 (17) |
| S1—C1 | 1.660 (3) | C2—C2$^{v}$ | 1.530 (3) |
| N1—C1 | 1.162 (3) | N2—H2 | 0.88 (4) |
| | | | |
| S1$^{i}$—Cu1—S1$^{ii}$ | 120.0 | C2$^{iv}$—N2—C2$^{iii}$ | 110.31 (10) |
| S1$^{ii}$—Cu1—S1 | 120.0 | C2$^{iii}$—N2—C2 | 110.31 (10) |
| S1$^{i}$—Cu1—S1 | 120.0 | N2—C2—C2$^{v}$ | 108.62 (10) |
| C1—S1—Cu1 | 107.85 (9) | C2$^{iv}$—N2—C2 | 110.31 (10) |
| N1—C1—S1 | 176.3 (2) | | |

Symmetry codes: (i) −x+y+1, −x+2, z; (ii) −y+2, x−y+1, z; (iii) −x+y+1, −x+1, z; (iv) −y+1, x−y, z; (v) x, y, −z+3/2.

**Table S3**. Selected geometrical parameters (distances/Å and angles/°) for **3**.

Geometric parameters (Å, °)

| | | | |
|---|---|---|---|
| Cu1—N1i | 1.9842 (16) | N4—H4 | 1.0000 |
| Cu1—N1 | 1.9842 (16) | N4—C6 | 1.489 (2) |
| Cu1—N2 | 1.9952 (16) | N4—C7 | 1.491 (2) |
| Cu1—N2i | 1.9953 (16) | N4—C8 | 1.494 (2) |
| S1—C1 | 1.6341 (19) | S3—O1 | 1.5132 (14) |
| S2—C2 | 1.633 (2) | S3—C9 | 1.776 (2) |
| N1—C1 | 1.155 (2) | S3—C10 | 1.785 (2) |
| N2—C2 | 1.159 (2) | C3—C8 | 1.538 (3) |

| | | | |
|---|---|---|---|
| N3—C3 | 1.464 (2) | C4—C7 | 1.538 (3) |
| N3—C4 | 1.466 (2) | C5—C6 | 1.528 (3) |
| N3—C5 | 1.468 (3) | | |
| | | | |
| N1i—Cu1—N1 | 180.0 | N1—Cu1—N2i | 88.23 (6) |
| N1i—Cu1—N2i | 91.77 (6) | N1—Cu1—N2 | 91.77 (6) |
| N1i—Cu1—N2 | 88.23 (6) | N2—Cu1—N2i | 180.0 |

Symmetry code: (i) −x+1, −y+1, −z+1.

**Table S4**. Selected geometrical parameters (distances/Å and angles/°) for **4**.

| | | | |
|---|---|---|---|
| S1—C1 | 1.6294 (18) | N3—C4i | 1.4665 (15) |
| N1—C1 | 1.164 (2) | N3—C4 | 1.4665 (15) |
| N2—C2 | 1.4909 (16) | N3—C5 | 1.468 (2) |
| N2—C2i | 1.4909 (16) | C2—C4 | 1.5317 (18) |
| N2—C3 | 1.495 (2) | C3—C5 | 1.535 (2) |
| | | | |
| N1—C1—S1 | 179.22 (17) | C4—N3—C5 | 108.92 (8) |
| C2—N2—C2i | 109.66 (15) | N2—C2—C4 | 108.04 (11) |
| C2—N2—C3 | 109.84 (10) | N2—C3—C5 | 107.95 (13) |
| C2i—N2—C3 | 109.84 (10) | N3—C4—C2 | 111.18 (10) |
| C4i—N3—C4 | 109.00 (13) | N3—C5—C3 | 111.06 (14) |
| C4i—N3—C5 | 108.92 (8) | | |

Symmetry code: (i) x, −y+1/2, z.

**Table S5**. Selected geometrical parameters (distances/Å and angles/°) for **5**.

| | | | |
|---|---|---|---|
| S1—C1 | 1.619 (2) | S2—C2 | 1.617 (2) |
| N1—C1 | 1.144 (3) | N3—C5 | 1.486 (3) |
| N2—C2 | 1.143 (3) | N4—C6 | 1.484 (3) |
| N3—C3 | 1.483 (3) | N4—C7 | 1.484 (3) |
| N3—C4 | 1.487 (2) | N4—C8 | 1.495 (2) |

| | | | |
|---|---|---|---|
| C4—C8 | 1.510 (3) | C5—C6 | 1.505 (3) |
| | | C3—C7 | 1.513 (3) |
| | | | |
| N1—C1—S1 | 179.7 (2) | N3—C5—C6 | 109.07 (16) |
| N2—C2—S2 | 178.7 (2) | N4—C7—C3 | 108.89 (15) |
| C3—N3—C5 | 109.72 (16) | N3—C3—C7 | 108.50 (16) |
| C4—N3—C3 | 110.47 (16) | N4—C8—C4 | 108.30 (15) |
| C4—N3—C5 | 110.28 (16) | N4—C6—C5 | 108.58 (15) |
| C6—N4—C7 | 110.22 (15) | N3—C4—C8 | 109.02 (15) |
| C6—N4—C8 | 110.19 (15) | C7—N4—C8 | 110.20 (16) |

**Table S6**. Selected geometrical parameters (distances/Å and angles/°) for **6**.

| | | | |
|---|---|---|---|
| S1—C1 | 1.643 (5) | N2—C3 | 1.493 (4) |
| N1—C1 | 1.154 (6) | C2—C2iii | 1.552 (10) |
| O1—H1 | 0.80 (6) | C3—C3iv | 1.530 (8) |
| O1—H1i | 0.80 (5) | N2—C3—C3iv | 108.8 (2) |
| N2—H2 | 1.0000 | C2—N2—C3ii | 109.9 (2) |
| N2—C2 | 1.489 (5) | C2—N2—C3 | 109.9 (2) |
| N2—C3ii | 1.493 (4) | C3ii—N2—C3 | 110.9 (4) |
| | | | |
| N1—C1—S1 | 179.5 (4) | N2—C2—C2iii | 108.5 (3) |

Symmetry codes: (i) −x+1, −y+1, z; (ii) x, −y+1, z; (iii) −x+2, −y+1, z; (iv) −x+2, y, z.

**Table S7**. Selected geometrical parameters (distances/Å and angles/°) for **7**.

| | | | |
|---|---|---|---|
| Cu1—O1 | 1.978 (2) | S1—C1 | 1.631 (3) |
| Cu1—O1$^{i}$ | 1.978 (2) | S2—O1 | 1.538 (2) |
| Cu1—N1 | 1.939 (2) | S2—C2 | 1.764 (3) |
| Cu1—N1i | 1.939 (2) | S2—C3 | 1.771 (3) |
| N1—C1 | 1.159 (4) | | |

| | | | |
|---|---|---|---|
| O1—Cu1—O1i | 180.0 | O1—S2—C2 | 103.28 (14) |
| N1i—Cu1—O1 | 92.55 (9) | O1—S2—C3 | 103.39 (14) |
| N1—Cu1—O1i | 92.55 (9) | C2—S2—C3 | 99.04 (19) |
| N1i—Cu1—O1i | 87.45 (9) | S2—O1—Cu1 | 120.75 (13) |
| N1—Cu1—O1 | 87.45 (9) | C1—N1—Cu1 | 167.4 (3) |
| N1—Cu1—N1i | 180.0 | N1—C1—S1 | 179.1 (3) |

Symmetry code: (i) −x+1, −y+1, −z+1.

**Table S8**. Hydrogen-bonds geometry (Å, °).

| D—H···A | D—H | H···A | D···A | D—H···A |
|---|---|---|---|---|
| **1** | | | | |
| N5—H5···N4$^{ii}$ | 1.00 | 1.69 | 2.683 (14) | 171 |
| **3** | | | | |
| N4—H4···O1$^{ii}$ | 1.00 | 1.77 | 2.680 (2) | 150 |
| **4** | | | | |
| N2—H2···N1 | 0.93 (3) | 1.83 (3) | 2.763 (2) | 180 (2) |
| **5** | | | | |
| N3—H3···N1$^{i}$ | 0.89 (2) | 1.80 (2) | 2.696 (2) | 176.2 (19) |
| N4—H4···N2$^{ii}$ | 0.96 (3) | 1.75 (3) | 2.696 (2) | 168 (2) |
| **6** | | | | |
| O1—H1···N1 | 0.80 (6) | 1.92 (6) | 2.720 (5) | 179 (6) |
| N2—H2···O1 | 1.00 | 1.80 | 2.724 (4) | 152 |

Symmetry codes: **1:** x, −y+1, z+1/2 (ii); **3:** −x+1/2, y−1/2, −z+3/2 (ii); **5:** −x+1, −y+1, −z+1 (i); x−1/2, −y+3/2, z−1/2 (ii).

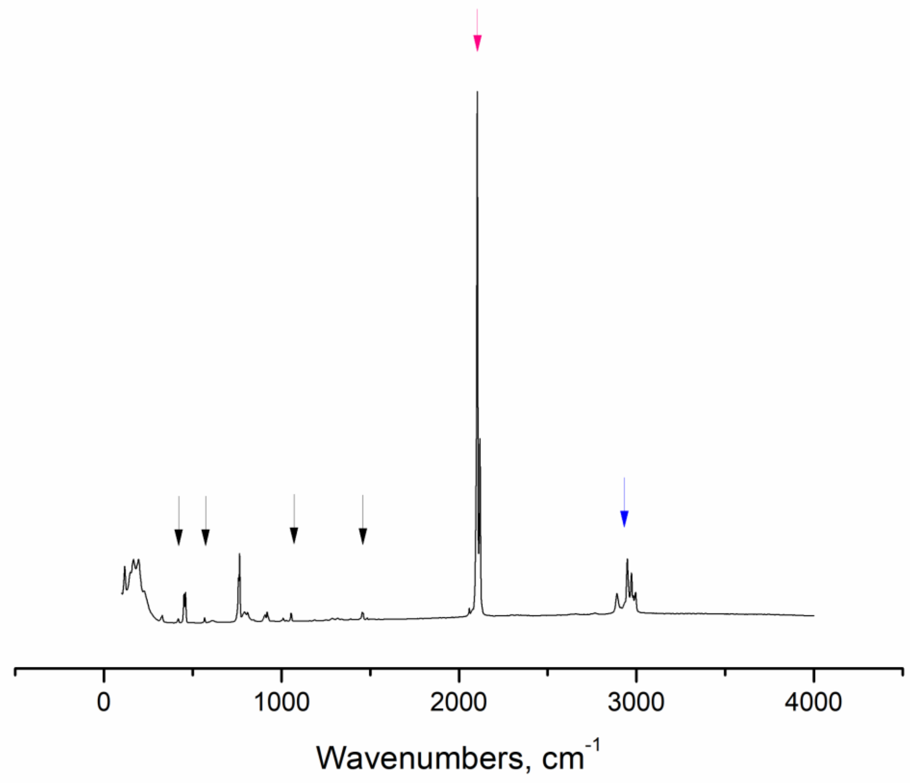


Figure S1. Raman spectrum of compound **1**. Black arrows indicate peaks arising from *dabco* moiety vibrations, red arrow shows the $\nu_1$ mode of $SCN^-$ anion, the region of C-H vibrations is marked by blue arrow.

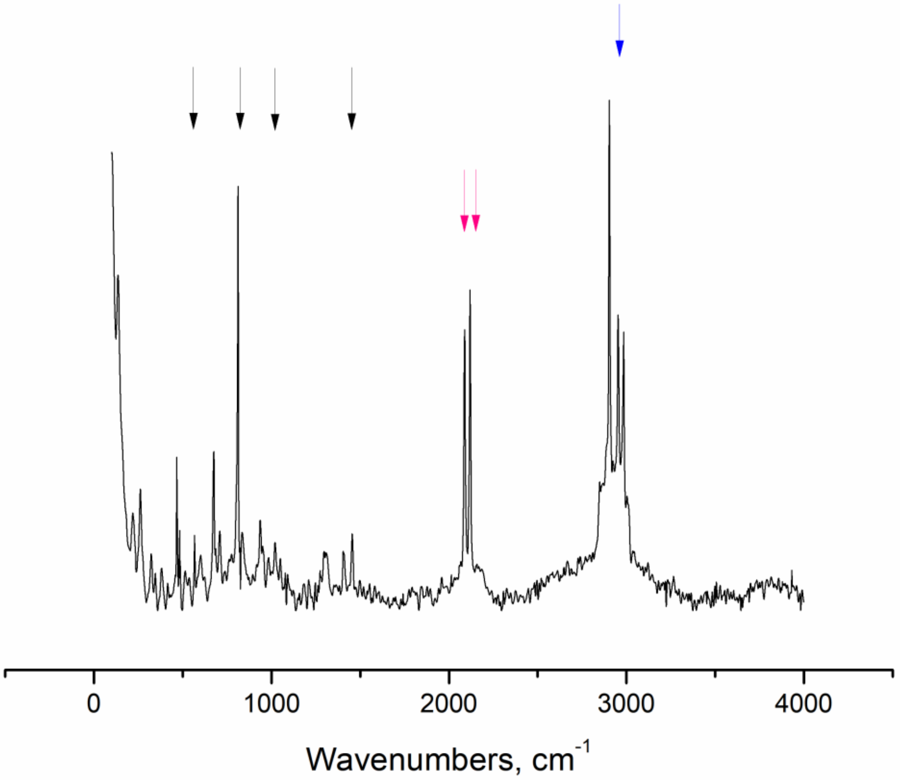


Figure S2. Raman spectrum of compound **3**. Black arrows indicate peaks arising from *dabco* moiety vibrations, red arrow shows the $\nu_1$ mode of $SCN^-$ anion, the region of C-H vibrations is marked by blue arrow.

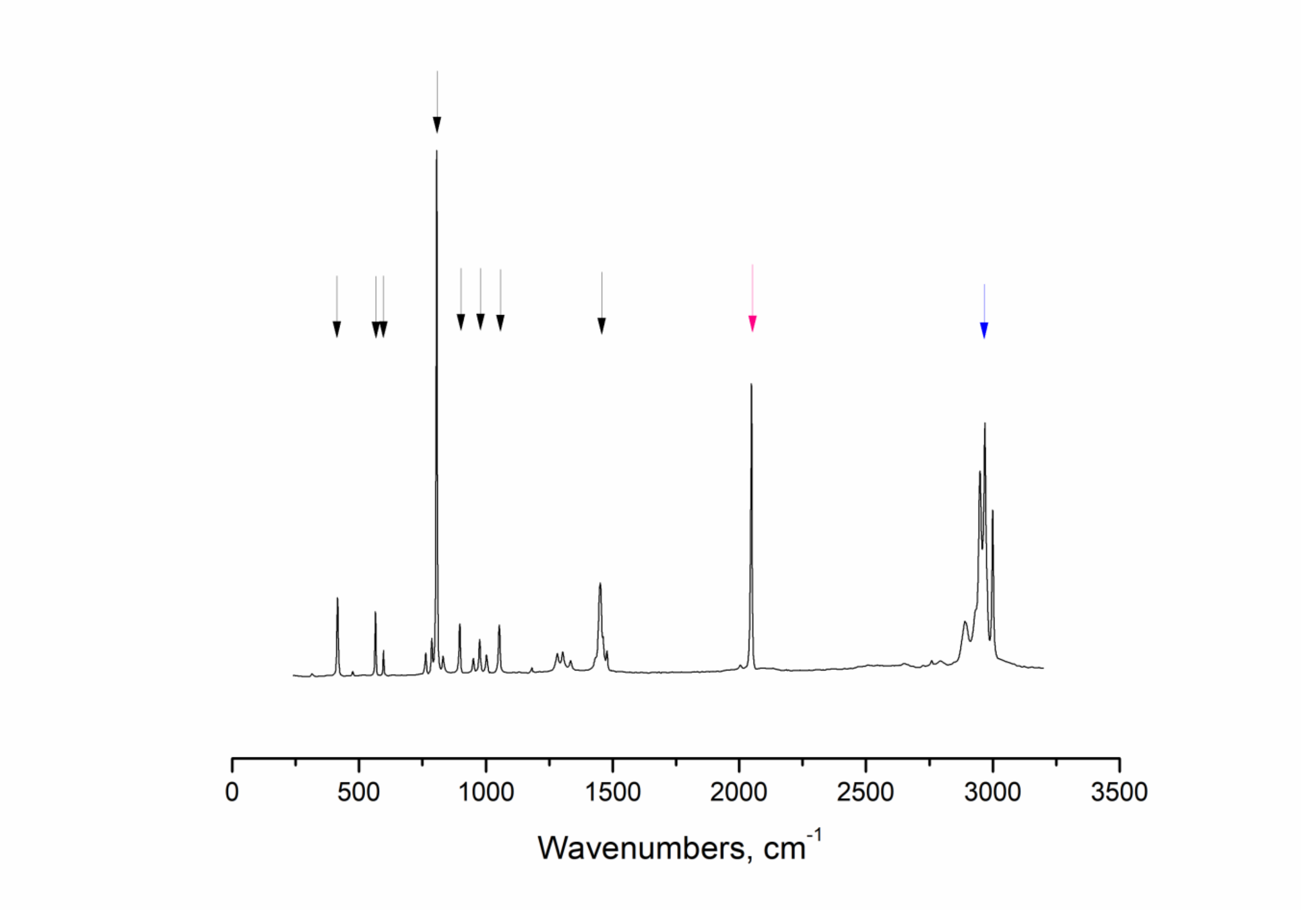


Figure S3. Raman spectrum of compound **4**. Black arrows indicate peaks arising from *dabco* moiety vibrations, red arrow shows the $\nu_1$ mode of $SCN^-$ anion, the region of C-H vibrations is marked by blue arrow.

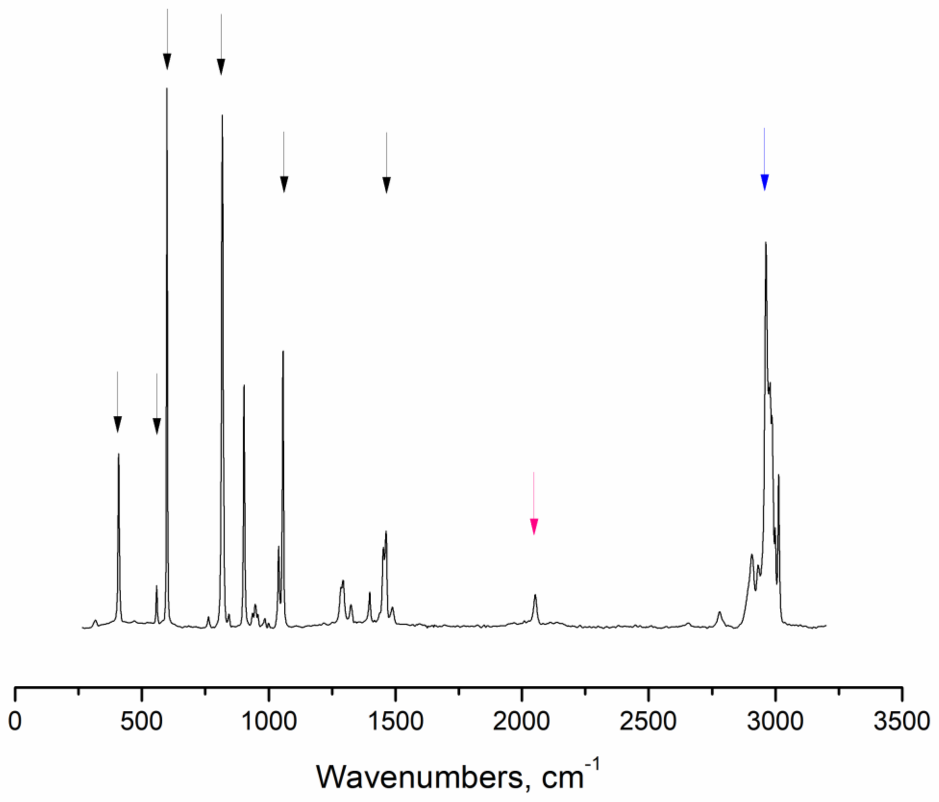


Figure S4. Raman spectrum of compound **5**. Black arrows indicate peaks arising from *dabco* moiety vibrations, red arrow shows the $\nu_1$ mode of $SCN^-$ anion, the region of C-H vibrations is marked by blue arrow.

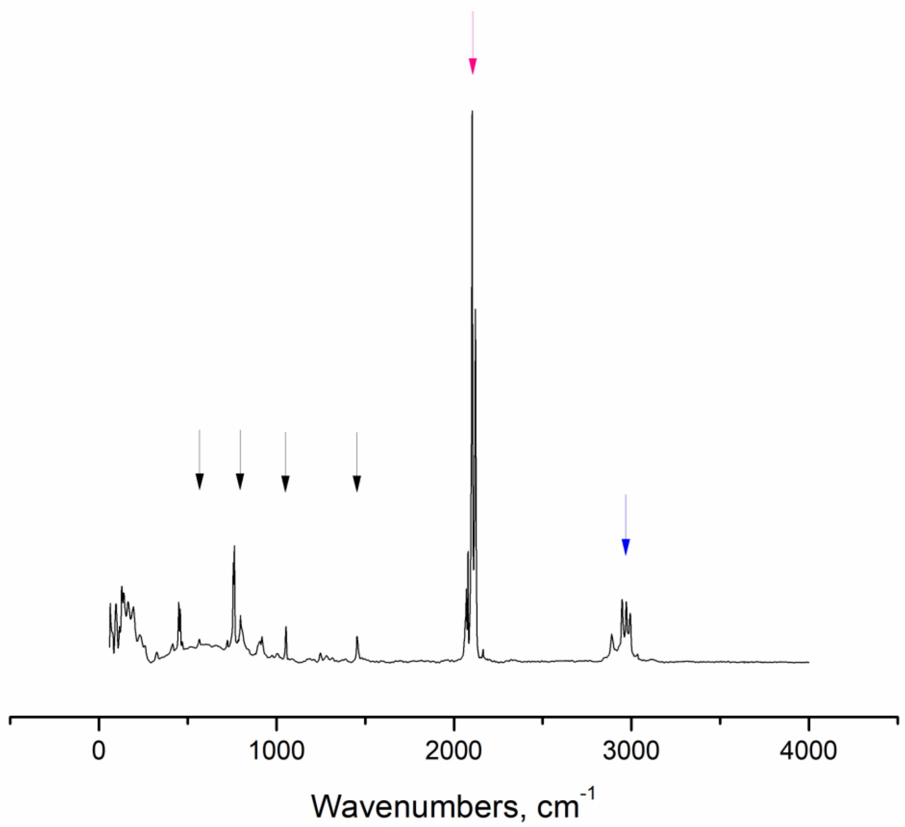


Figure S5. Raman spectrum of compound **6**. Black arrows indicate peaks arising from *dabco* moiety vibrations, red arrow shows the $\nu_1$ mode of $SCN^-$ anion, the region of C-H vibrations is marked by blue arrow.

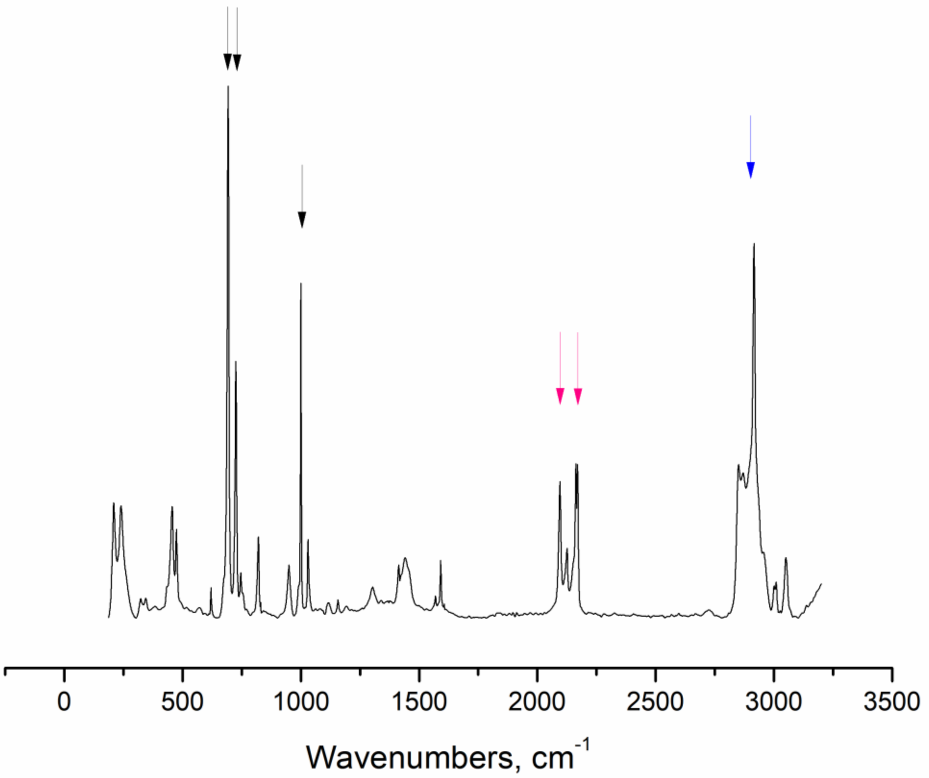


Figure S6. Raman spectrum of compound **7**. Black arrows indicate peaks arising C-S (lower wavenumbers) and S=O vibrations, red arrow shows the $\nu_1$ mode of $SCN^-$ anion, the region of C-H vibrations is marked by blue arrow.

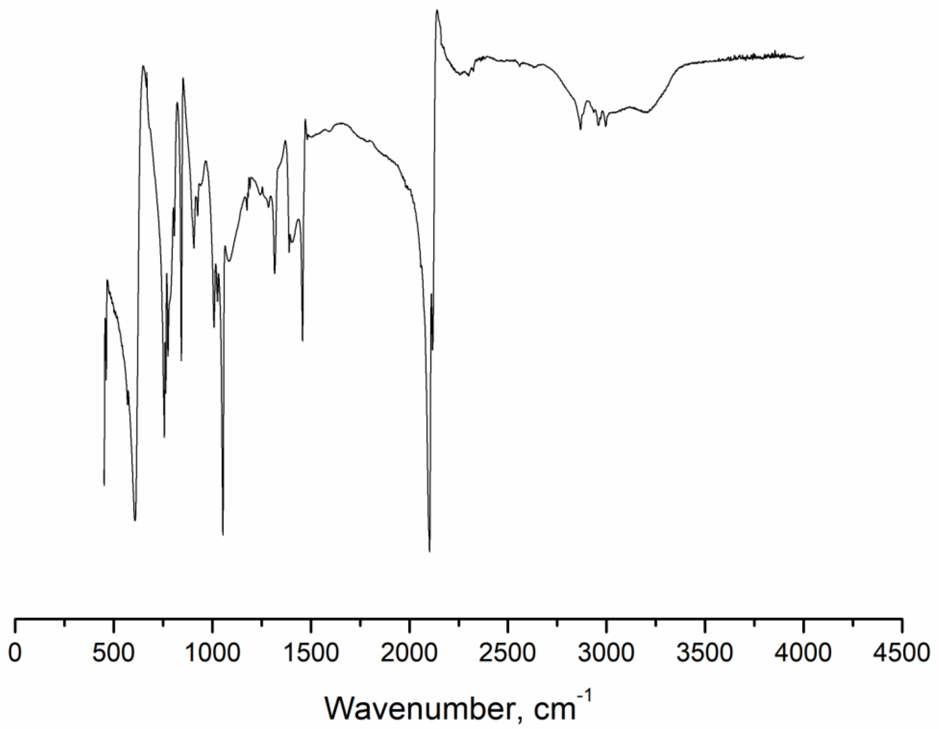


Figure S7. IR spectrum of compound **1**.

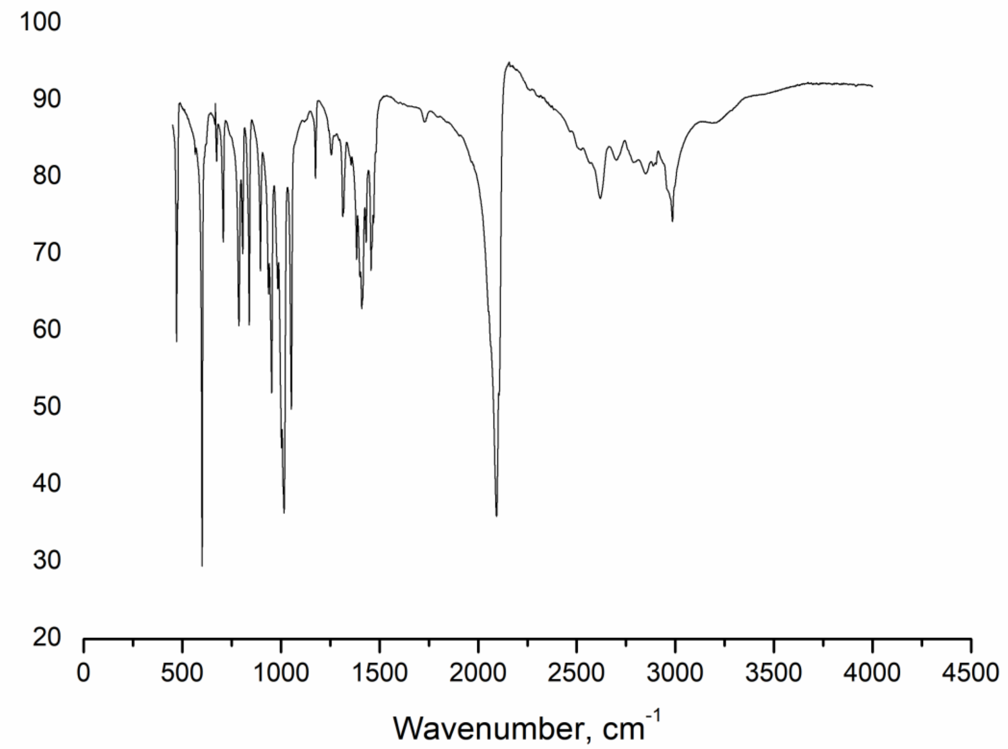


Figure S8. IR spectrum of compound **3**.

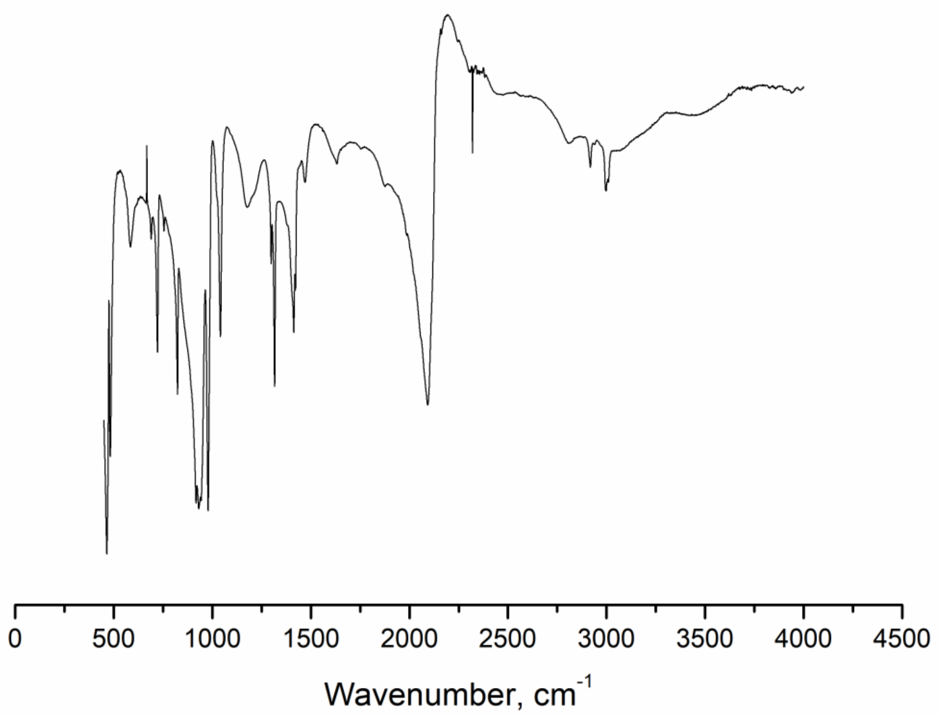


Figure S7. IR spectrum of compound **4**.
